# Integrative Transcriptomic and Proteomic Analysis Reveals an Alternative Molecular Network of Glutamine Synthetase 2 Corresponding to Nitrogen Deficiency in Rice (*Oryza sativa* L.)

**DOI:** 10.3390/ijms22147674

**Published:** 2021-07-18

**Authors:** Ting Liang, Zhengqing Yuan, Lu Fu, Menghan Zhu, Xiaoyun Luo, Wuwu Xu, Huanran Yuan, Renshan Zhu, Zhongli Hu, Xianting Wu

**Affiliations:** 1 State Key Laboratory of Hybrid Rice, Wuhan University, Wuhan 430072, China; 2015202040066@whu.edu.cn (T.L.); 2016202040066@whu.edu.cn (Z.Y.); 2017282040173@whu.edu.cn (L.F.); 2020202040207@whu.edu.cn (M.Z.); 2015202040067@whu.edu.cn (X.L.); xuwuwu@whu.edu.cn (W.X.); huanranyuan@whu.edu.cn (H.Y.); renshan8@whu.edu.cn (R.Z.); huzhongli@whu.edu.cn (Z.H.); 2 College of Life Sciences, Wuhan University, Wuhan 430072, China; 3 Crop Research Institute, Sichuan Academy of Agricultural Science, Chengdu 610000, China

**Keywords:** rice, root system architecture, transcriptome, proteome, N availability

## Abstract

Nitrogen (N) is an essential nutrient for plant growth and development. The root system architecture is a highly regulated morphological system, which is sensitive to the availability of nutrients, such as N. Phenotypic characterization of roots from LY9348 (a rice variety with high nitrogen use efficiency (NUE)) treated with 0.725 mM NH_4_NO_3_ (1/4N) was remarkable, especially primary root (PR) elongation, which was the highest. A comprehensive analysis was performed for transcriptome and proteome profiling of LY9348 roots between 1/4N and 2.9 mM NH_4_NO_3_ (1N) treatments. The results indicated 3908 differential expression genes (DEGs; 2569 upregulated and 1339 downregulated) and 411 differential abundance proteins (DAPs; 192 upregulated and 219 downregulated). Among all DAPs in the proteome, glutamine synthetase (GS2), a chloroplastic ammonium assimilation protein, was the most upregulated protein identified. The unexpected concentration of GS2 from the shoot to the root in the 1/4N treatment indicated that the presence of an alternative pathway of N assimilation regulated by GS2 in LY9348 corresponded to the low N signal, which was supported by GS enzyme activity and glutamine/glutamate (Gln/Glu) contents analysis. In addition, N transporters (*NRT2.1*, *NRT2.2*, *NRT2.3*, *NRT2.4*, *NAR2.1*, *AMT1.3*, *AMT1.2*, and putative *AMT3.3*) and N assimilators (*NR2*, *GS1;1*, *GS1;2*, *GS1;3*, *NADH-GOGAT2*, and *AS2*) were significantly induced during the long-term N-deficiency response at the transcription level (14 days). Moreover, the Kyoto Encyclopedia of Genes and Genomes (KEGG) pathway analysis demonstrated that phenylpropanoid biosynthesis and glutathione metabolism were significantly modulated by N deficiency. Notably, many transcription factors and plant hormones were found to participate in root morphological adaptation. In conclusion, our study provides valuable information to further understand the response of rice roots to N-deficiency stress.

## 1. Introduction

Rice (*Oryza sativa* L.) is a main crop in many countries worldwide [1], including China. Specifically, it contributes to 43.7% and 36.9% of the total grain output in China and worldwide, respectively [2]. In China, rice accounts for 22.8% of the world’s total rice cultivation area [3]. Moreover, hybrid rice cultivation has significantly increased yield to feed the large population of China [4]. Honglian-type cytoplasmic sterility (CMS) is one of the three major CMS for three-line hybrid rice generation, and the hybrid rice variety LY9348 produced by Honglian-type male sterile line Longhong4A demonstrates remarkable agricultural characteristics, such as high yield, broad adaptability, disease resistance, insect resistance, high temperature tolerance, and high nitrogen use efficiency (NUE) [5,6].

Nitrogen (N), one of the essential nutrient elements for plant growth and yield [7,8], is not only a key component for building up many cell functional molecules, such as nucleic acid, ATP, amino acid, chlorophyll, and various plant hormones, but also a key regulator of many biological processes, including amino acid metabolism, carbon metabolism, and protein synthesis [9]. Furthermore, if used as the main fertilizer factor to increase rice yield, N fertilizer plays an essential role in guaranteeing the high and stable rice production in China annually. The average amount of fertilizer applied in China is reported to be 180 kg/hm^2^, which is about 75% higher than the world average of 102 kg/hm^2^ [10]. However, the NUE in the rice fields of China is only about 30%, far lower than the world average.

In the paddy field, N exists in two forms: ammonium (NH_4_^+^) and nitrate (NO_3_^–^). Of these, rice prefers ammonium over nitrate as its N source [11]. In flooded wetlands or acidic soils, NH_4_^+^ is the main N form, whereas, in well-drained soils, NH_4_^+^ converts rapidly to nitrite (NO_2_^–^) and then to NO_3_^–^. Thus, the main form of N in upland soils is NO_3_^–^, regardless of the form of N fertilizer applied. For most plants, some NO_3_^–^ can be absorbed by nitrate transporters (NRTs) and further assimilated at the root. However, in its major pathway, NO_3_^–^ is transported to the stem, where it is first reduced by nitrate reductase in the cytoplasm and then to NH_4_^+^ by nitrite reductase in the plastids. Then, it is assimilated by glutamine synthetase (GS), both in the cytoplasm and the plastids. NH_4_^+^, which is either generated from NO_3_^–^ or is directly absorbed by ammonium transporters, can further be assimilated into amino acids and circulated through the GS/glutamate synthase (GS/GOGAT) cycle. The major GS/GOGAT isozymes are chloroplastic glutamine synthetase 2 (GS2) and Fd-GOGAT, as well as cytoplasmic glutamine synthetase 1 (GS1) and NADH-GOGAT, which are the key enzymes catalyzing the conversion of NH_4_^+^ and glutamate to glutamine [12,13,14,15,16,17,18,19]. Thereafter, glutamate can be transformed to other amino acids by different aminotransferases. Asparagine synthetase (AS) catalyzes glutamine to form asparagine and glutamate. Together, AS and GS are considered to be crucial in primary N metabolism [20,21]. In addition, mitochondrial glutamate dehydrogenase (GDH) can integrate NH_4_^+^ into glutamate in response to low NH_4_^+^ supply [22,23]. So far, the genes related to N metabolism of rice have been reported; these genes include *OsNRT1.1B* [24], *OsNRT1.1A* [25], *OsNRT2.2* [26], *OsNRT2.3a* [26], *OsNRT2.4* [27], *OsNRT2.3b* [28], *OsAMT* [29], *OsNR2* [30], *OsNLP4–OsNiR* cascade [31], *OsGS1/2* [32,33], *OsGOGAT* [34], the *OsMYB61/OsGRF4* cascade [35], *OsNAC42* [36], and *OsTCP19* [37], and they have laid a solid foundation for high NUE study and selection in rice breeding programs. However, although *GS1* overexpression can elevate NUE in rice, the understanding of the underlying network remains unclear [14,38,39,40]. Analyzing the relevant regulatory network and cultivating nitrogen-sensitive high-yield varieties (high yield at moderate or even lower N input conditions) are of great significance for improving the rice crop yield and NUE, reducing fertilizer input in agricultural production, and realizing the sustainable development of agriculture [4,41,42].

In rice, the root system is the main N-absorbing organ. The root-specific phenotypes, including root morphology, root–shoot ratio (RSR), root vigor, and root length density, are important indexes for screening rice varieties with variable NUE and providing important information to evaluate N sensing and absorption in rice [23,43]. After NO_3_^–^ is absorbed into the root epidermal and cortical cells, it is reduced and stored in vacuoles and then transported through the xylem across long distances up to the leaves. NO_3_^–^ assimilation accounts for a large proportion in the root system under low N concentration. Only when the assimilating capacity of the root is overloaded does a small quantity of NO_3_^–^ become transported to the shoot via the xylem [44,45]. Under low N stress, plant hormones and the nitric oxide signaling pathway have synergistic or antagonistic effects on root elongation in crops [46]. Root morphology changes can shape the plant underground system such that it adapts to soil nutrient availability [47]. Studies have shown that the root system architecture had an important effect on the grain yield of crops grown under high and low N conditions and demonstrated the underlying importance of root traits in NUE [48].

Transcriptomics and proteomics are majorly used for analyzing certain data profiles to provide a better understanding of abiotic stress in plants [49,50]. Through synchronization detections on mRNA and protein changes in samples and data mining through the two integrated and compared data sheets, the regular pattern and nature of life activities at both transcription and translation level can be explored [51]. Moreover, this methodology aids in revealing the reciprocal regulation effects or correlations between the two levels. For example, the connection networks between genes and proteins in rice are conducted on the responses and mechanisms of two-line hybrid rice variety Wuxiang S (WXS) during its fertility transition [52]. In a previous study, RNA sequencing (RNA-Seq) analysis of rice roots and shoots under N-free and high-NH_4_^+^ conditions was performed, and a list of candidate genes related to NH_4_^+^ response and metabolisms was obtained [53]. However, few high-NUE rice varieties have been reported thus far, and almost no research has focused on the responses of higher NUE rice to N supply through an integrated transcriptomic and proteomic analysis. Therefore, by analyzing the high-NUE rice variety LY9348 roots’ responses in different N hydroponics experiments with six N doses at the seedling stages, we adapt the omics profiling to determine the morphological and physiological characteristics related to the high-NUE rice. The integrated transcriptomic and proteomic analysis among the two selected treatments (1/4N (0.725 mM NH_4_NO_3_) vs. 1N (2.9 mM NH_4_NO_3_)) may provide a deeper understanding of the differences in the regulatory network of the roots responding to N-foraging signal transduction in NUE rice after a N-deficient and N-sufficient nutrient supply.

## 2. Results

### 2.1. Root Phenotypes Identified in Different Nitrogen Dosage Treatments

**Figure 1:**
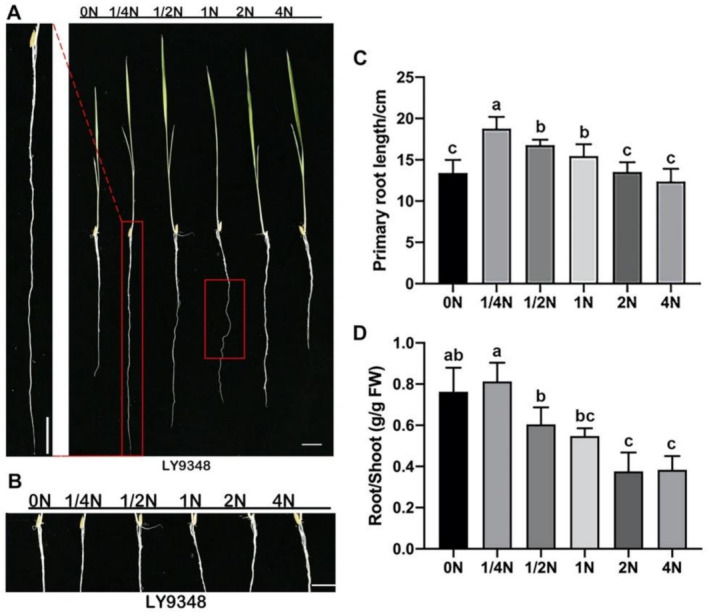
Phenotypic characteristics of LY9348 after different N dosage treatments. (**A**) Morphological characteristics of LY9348 at six N concentrations; (**B**) characteristics of LRs of LY9348 at six N concentrations; (**C**) PR length of LY9348 at six N concentrations; (**D**) the root-to-shoot ratio in fresh weight (FW g/g) at six N concentrations. Bars represent the standard error (SE; *n* = 3). Different letters indicate significant differences (*p* < 0.05, one-way ANOVA, Tukey’s HSD test). Scale length = 1 cm.

NH_4_NO_3_ provides ammonium and nitrate at a 1:1 ratio as the N source in standard hydroponic culture solution. To determine the deficient N concentration for LY9348, six different N concentrations were applied in the experiment. With 2.9 mM NH_4_NO_3_ set as the 1N treatment, the other five treatments were 0N (no NH_4_NO_3_), 1/4N (0.725 mM NH_4_NO_3_), 1/2N (1.45 mM NH_4_NO_3_), 2N (5.8 mM NH_4_NO_3_), and 4N (11.6 mM NH_4_NO_3_). After treatment for 14 days, primary root (PR) elongation—the N-deficient phenotype—was observed to be the highest for the 1/4N treatment in LY9348, followed by 1/2N and 1N treatments. In addition, PR elongation was the lowest with the 0N and 4N treatments (Figure 1A,C), which indicated that PR-inhibited phenotypes were identified under severe N deficiency (0N) and under oversupplied N (4N) conditions in LY9348. Notably, lateral root (LR) initiation and elongation—another N-deficient phenotype—were inhibited in LY9348 for all six N treatments (Figure 1B).

Additionally, plants have evolved various strategies for survival when grown under nutrient deficiency environments, including an increase in the root-to-shoot ratio [54]. Compared with other N treatments, LY9348 with 1/4N treatment showed a significantly increased root-to-shoot ratio under N-deficiency conditions (Figure 1D), mainly resulting from increased root biomass (Supplementary Materials Figure S1A) but the unaffected shoot biomass (Supplementary Materials Figure S1B).

Moreover, the helical root phenotype was induced after treatment with >2.5 mM NH_4_NO_3_ in the agar medium; this was a sign for ammonium-induced low acidification responses in Nipponbare [55]. Helical root phenotype was also observed after 1N treatment in LY9348, but not after the other two higher N treatments, 2N and 4N (Figure 1A). Therefore, 1/4N was set as the N-deficient treatment and 1N was set as the N-sufficient treatment for LY9348.

### 2.2. N-Deficiency Responses in Proteome Profiling in LY9348 Roots

**Figure 2:**
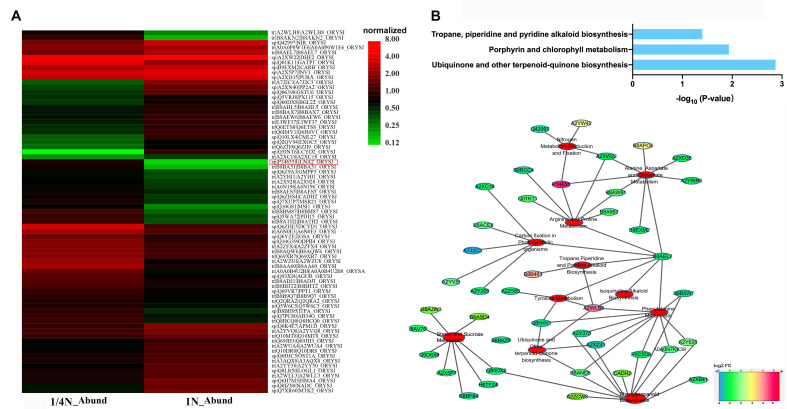
N-deficiency responses in proteome profiling in LY9348 roots. (**A**) Heat map analysis of candidate DAPs; (**B**) cluster of KEGG pathways and DAPs putatively related to candidate pathways. Networks were visualized by Cytoscape (3.2.0). DAPs: different abundance proteins; Abund: quantitative protein abundance value of sample; FC: fold change.

To detect the protein network underlying N-deficiency signals and responses of the high-NUE rice variety LY9348, 14-day roots of the rice treated with 1/4N and 1N were collected for proteomic analysis. Label-free liquid chromatography–mass spectrometry (LC–MS) analysis was applied to identify and quantify differential abundance proteins (DAPs) from these two N treatments. Accordingly, 35,279 and 35,896 spectra were generated, and 11,030 and 11,618 unique peptides and 2273 and 2354 proteins were identified, with a false discovery rate (FDR) of ≤ 1% in LY9348 roots with 1/4N and 1N treatments, respectively (Supplementary Materials Table S1). Of these proteins, 441 DAPs were identified in 1/4N and 1N treatments (1/4N_1N), with a |fold-change (FC)| of ≥2 and a *p* value of <0.05. Moreover, 192 and 219 DAPs were more and less abundant in 1/4N treatment than in 1N treatment, respectively. The candidate known function DAPs of interest were selected for cluster analysis (Supplementary Materials Table S2; Figure 2A). Of these DAPs, glutamine synthetase (P14655), annotated as *OsGS2* (LOC_Os04g56400) in the rice genome, was the most upregulated protein identified in LY9348 roots with 1/4N treatment.

To further illustrate the functions of DAPs, we performed pathway enrichment analysis of DAPs in 1/4N_1N based on the Kyoto Encyclopedia of Genes and Genomes (KEGG) database (Figure 2B). In total, 18 DAPs were significantly enriched in three pathways (corrected *p* value < 0.05): (1) ubiquinone and other terpenoid-quinone biosynthesis; (2) porphyrin and chlorophyll metabolism pathway; and (3) tropane, piperidine, and pyridine alkaloid biosynthesis. Moreover, in the analysis of the number of DAPs in KEGG pathways, these pathways were the most significant: phenylpropanoid biosynthesis (17 DAPs); phenylalanine metabolism (16 DAPs); oxidative phosphorylation (14 DAPs); glycolysis/gluconeogenesis (13 DAPs); starch and sucrose metabolism (13 DAPs); glutathione (GSH) metabolism (12 DAPs); and alanine, aspartate, and glutamate metabolism (10 DAPs; Supplementary Materials Table S3).

To obtain an overview on the proteomic adaptation of LY9348 roots under N-deficient conditions, we functionally integrated pathways with a higher number of DAPs known to be typical N-response-related pathways and this significantly enriched three pathways via the DAPs (Figure 2B). We observed that the highest upregulation of GS2 (P14655) acted as a hub to correlate N metabolism with arginine and proline metabolism, as well as alanine, aspartate, and glutamate metabolism. Simultaneously, the pathway of phenylalanine metabolism was associated with ubiquinone and other terpenoid-quinone biosynthesis through the upregulated protein (amino transferase, A2WLB8), which is annotated as *DNR1* (LOC_Os01g08270), related to nitrogen use efficiency (NUE) in the rice genome. The result demonstrated a cross-network relationship between the DAPs in the candidate pathways in response to low N.

### 2.3. GS2 Unexpected Concentration in the Roots after 1/4N Treatment

**Figure 3:**
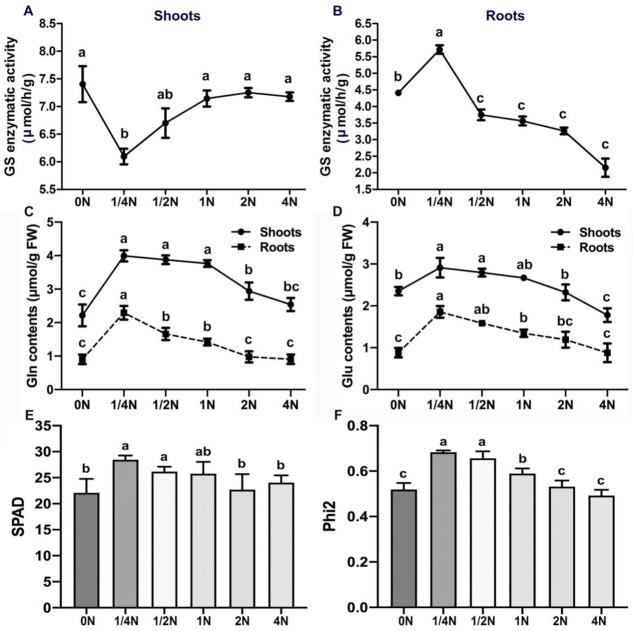
Physiological and biochemical characteristics of LY9348 after different N dosage treatments. (**A**,**B**) The GS enzyme activity (μmol/h/g) of LY9348 in the shoots (**A**) and in the roots (**B**) grown hydroponically for 14 days at six N concentrations; (**C**,**D**) the change of Gln (**C**) and Glu (**D**) contents (μmol/g) in LY9348 shoots and roots at six N concentrations; (**E**) relative chlorophyll content (SPAD) of LY9348 at six N concentrations; (**F**) photosynthesis rate (Phi2) of LY9348 at six N concentrations. Bars represent the standard error (SE; *n* = 3). Different letters indicate significant differences (*p* < 0.05, one-way ANOVA, Tukey’s HSD test).

In previous studies, GS2 was found to be the larger isoform of GS, localized in the chloroplast and responsible for reassimilating NH_4_^+^ released during photorespiration. It was also found in plastids for reducing NO_3_^−^ [12,13,14,15,16,17,18]. Cytosolic GS as GS1;2 (LOC_Os03g12290) was found to be mainly expressed in the roots [19]. To determine whether GS2 is highly abundant in LY9348 roots after 1/4N treatment, GS enzyme activity was analyzed for all six N treatments in 0.1 g of LY9348 shoot and root tissues, individually. GS activity significantly decreased from 7.14 μmol/h/g after 1N treatment to 6.10 μmol/h/g after 1/4N treatment in the shoots (Figure 3A). In contrast, GS activity apparently increased from 3.56 μmol/h/g after 1N treatment to 5.72 μmol/h/g after 1/4N treatment in the roots (Figure 3B). Because no obvious GS1;2 and GS1;1 protein accumulation was detected either in the roots or the shoots, the reduction of GS enzyme activity in the leaves (GS1;1 + GS2↓) and the increment of GS enzyme activity in the roots (GS1;2 + GS2↑) were potentially caused by the unexpected concentration of GS2 in the roots; therefore, the source of GS2 may have been the shoots. Consistent with the proteomic analysis, the opposite activity of GS enzyme in the shoots and roots was only observed as specific N-deficiency responses in LY9348 after 1/4N treatment, but not after other N treatments.

Next, the changes in Gln and Glu contents in the LY9348 shoots and roots were analyzed for all six N treatments by using content assay kits (Figure 3C,D). The Gln content was also found to increase from 1.42 μmol/g after 1N treatment to 2.29 μmol/g after 1/4N treatment in the roots, but no such obvious changes in the Gln content were noted between the 1/4N and 1N treatments in the shoots. Moreover, the trend of changes in the Glu content was similar to that of changes in the Gln content. These results indicated that, according to the upregulation of GS enzyme activity in the roots, significantly higher Gln levels were detectd in the roots as expected, and sufficient Glu molecules were supplied for Gln recycling, both in the shoots and roots under N-deficiency stress.

Since the biomass, and Gln and Glu contents of shoots were observed to be unaffected after the N-deficient (1/4N) treatment (Supplementary Materials Figure S1B, Figure 3C,D), leaf relative chlorophyll content (denoted by soil plant analysis development (SPAD) value) and photosynthesis rate (denoted by Phi2 value) were measured after all N treatments in LY9348. All the highest reads for SPAD (Figure 3E) and Phi2 (Figure 3F) values were also detected in 1/4N treatment, suggesting that the growth of shoots was stimulated, not inhibited, in N-deficient conditions in the high-NUE rice variety LY9348.

### 2.4. N-Deficiency Responses in Transcriptome Profiling in LY9348 Roots

**Figure 4:**
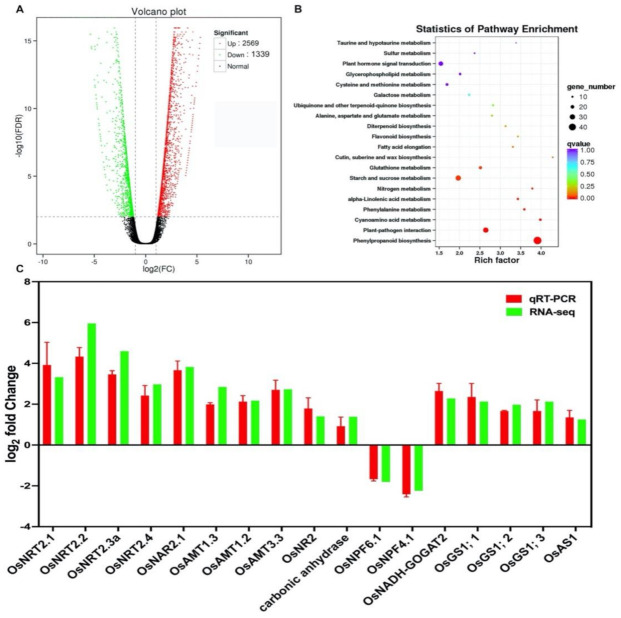
N-deficiency responses in transcriptome profiling in LY9348 roots. (**A**) Volcano plot of DEGs; (**B**) KEEG pathway of DEGs identified in LY9348 roots. The pathway names are provided in the vertical axis, rich factor in the horizontal axis, the size of the point represents the number of DEGs, and the color of the dot represents the q value; (**C**) quantitative real-time PCR (qRT-PCR) confirmation of selected genes. The expression level of each gene was normalized by using a reference gene *OsActin* and then calculated as relative expression level between 1/4N and 1N (control). Bars represent mean log_2_ fold change. KEGG: Kyoto Encyclopedia of Genes and Genomes; DEGs: different expression genes.

To determine the transcriptomic responses to N-deficiency signals in LY9348, 14-day roots of the plant exposed to 1/4N and 1N treatments were collected for transcriptomic analysis. As shown in the volcano plot, different expression genes (DEGs) were selected if their FDR < 0.01 and |log_2_FC| > 1. On comparing the results for 1/4N and 1N treatments, 3908 DEGs were detected in the roots of LY9348. Of these, 2569 were upregulated and 1339 genes were downregulated in the N-deficient (1/4N) condition (Supplementary Materials Table S4; Figure 4A).

KEGG enrichment analysis of DEGs identified in LY9348 roots under hydroponic conditions with 1/4N and 1N treatements is represented in Figure 4B. DEGs were noted to be significantly enriched in 20 pathways (corrected *p* value < 0.05). Of these, the pathways with the highest number of affected genes were phenylpropanoid biosynthesis (54 DEGs), plant hormone signal transduction (39 DEGs), starch and sucrose metabolism (37 DEGs), plant–pathogen interaction (34 DEGs), photosynthesis (23 DEGs), and glutathione metabolism (20 DEGs; Supplementary Materials Table S5).

To validate our RNA-Seq data profiling results, qRT-PCR was performed for 17 known root N absorption, transport, and assimilation genes (Supplementary Materials Table S6; Figure 4C). Moreover, the correlation analysis between qRT-PCR data and RNA-Seq gene expression levels was performed (Supplementary Materials Figure S2), and the result (R^2^ = 0.95) indicated that the gene expression data obtained from RNA-Seq was reliable.

### 2.5. Correlation Analysis between Proteome and Transcriptome

To compare and understand our transcriptomic and proteomic data, we performed correlation analysis, and the 1/4N–1N comparison yielded a total of 489 matched proteins and 771 matched genes (Supplementary Materials Table S7). Pearson correlation analysis was then used to separate the matched protein based on the distribution of the mRNA–protein ratio between the transcriptome and proteome into nine categories: no significant changes (com_normal, 368); upregulated (com_up, 2) and downregulated (com_down, 2) in both data profiling; no change in proteome but upregulated (*p*-normal t-up, 56) or downregulated (*p*-normal t-down, 7) in transcriptome or vice versa (t-normal *p*-up, 22 or t-normal *p*-down, 25); upregulated in proteome but downregulated in transcriptome (*p*-up t-down, 2) or vice versa (*p*-down t-up, 5). These nine categories were also present in the nine-image scatter plot for log_2_FC (Supplementary Materials Figure S3), and the correlation coefficient between the transcriptome and proteome was only 0.053, indicating a large disparity in relevance between genes transcribed and protein synthesized. Notably, of the genes with changes in mRNA level and protein abundance, ferredoxin–nitrite reductase (Q42997, LOC_Os02g52730) and GSH S-transferase (Q06398, LOC_Os01g37750) showed upregulated mRNA levels, but downregulated protein abundance (Table 1).

**Figure 5:**
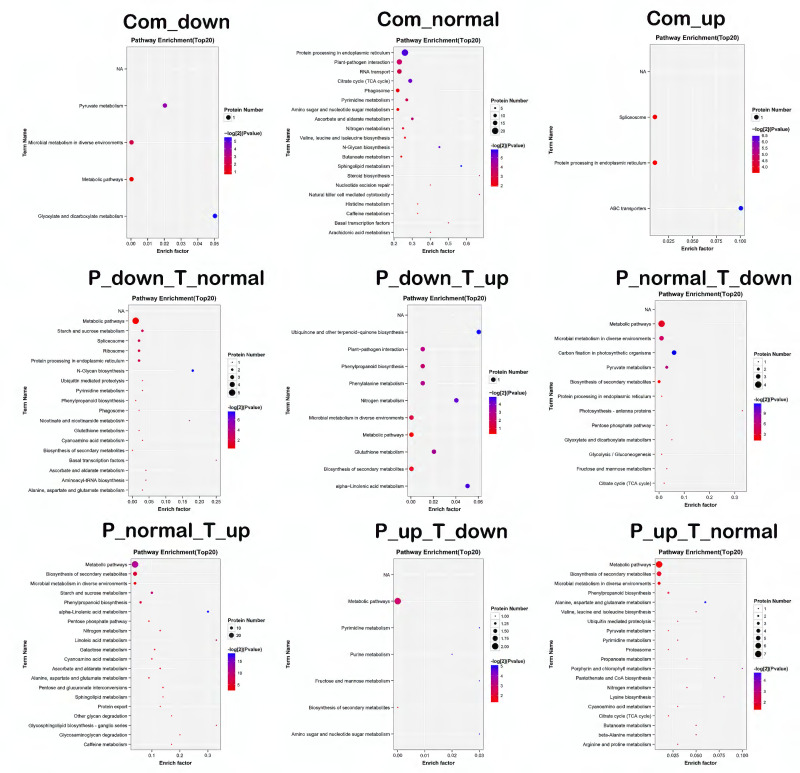
Correlation analysis of KEGG pathway enrichment bubble diagram. X axis for the enrichment factor; Y axis for KEGG pathway. The size of the bubbles represents the number of proteins on the annotated KEGG pathway; the color represents enrichment −log_2_(*p* value). A higher value indicates a higher significance.

Gene Ontology (GO) annotation indicated a large difference between the enriched GO terms of DAPs and that of DEGs; the DEGs were enriched in the extracellular region, antioxidant activity, transporter activity, signaling, and response to stimulus, whereas the DAPs were enriched in the membrane part, catalytic activities, and metabolic processes (Supplementary Materials Figure S4). KEGG pathway enrichment analysis was also performed in the nine categories to understand the functions associated with DEGs and DAPs (Figure 5). Genes and proteins that were downregulated together were annotated into four KEGG pathways, including glyoxylate and dicarboxylate metabolism and pyruvate metabolism. The jointly upregulated genes and proteins were annotated into three KEGG pathways, including protein procession in endoplasmic reticulum and the ABC transporter pathway. Of the KEGG pathways, the following pathways contained a higher abundance of proteins than of mRNAs: carbon fixation in photosynthesis organization; alanine, aspartate, and glutamate metabolism; and phenylpropanoid biosynthesis. In contrast, the following pathways had a lower abundance of proteins than of mRNAs: secondary metabolite biosynthesis, and starch and sucrose metabolism.

### 2.6. Modulation of Phenylpropanoid Biosynthesis Pathways under N-Deficiency Stress

Phenylpropanoid biosynthesis was found as the top regulated pathway, both in transcriptome and proteome in the KEGG analysis [56]. Of this pathway, 54 DEGs and 17 DAPs were selected and listed (Supplementary Materials Table S8; Table 2 and Table 3). We then annotated 20 peroxidases (14 upregulated and 6 downregulated) from 54 DEGs, and 4 peroxidase precursors (two upregulated and two downregulated) from 17 DAPs. The peroxidase multigenic family encodes secreted glycoproteins involved in the mechanisms of cell elongation, cell wall construction and differentiation, as well as pathogen defense response [57]. In total, 138 peroxidase genes have been annotated into the rice genome, but their functions are unclear. In this study, 24 peroxidase genes were altered due to the N-deficiency stress. In total, 10 β-glucosidase-like proteins (nine upregulated and one downregulated) from 54 DEGs, and 1 β-glucosidase homolog (downregulated) from 17 DAPs were observed. Of these, Os4BGlu12 was identified as a wound-induced protein, in response to herbivore attack and salinity stress [58]. The reason for this observation is the β-glucosidases are active in many metabolism processes, such as hydrolysis of cell-wall-derived oligosaccharides, as well as phytohormone regulation and lignification [59,60].

The 4-coumarate coenzyme A ligase (4CL) family members, such as *Os4CL1*, *Os4CL4*, and *Os4CL5*—the key enzymes for monolignol biosynthesis—were also observed. For example, Os4CL5 catalyzes lignin subunits G/S formation [61]. OsCAD2 (Q6ZHS4) was the upregulated DAP that stimulates the last step of monolignol biosynthesis [62]. Therefore, lignin formation and cell wall modification are highly regulated as a result of root morphological changes in response to N starvation signals [63].

The phenylalanine ammonia lyase (PAL) family genes are known to be upregulated to broaden the defenses toward varied rice pathogenic diseases, such as bacterial blight and sheath blight [64]. Notably, *OsPAL3*, *OsPAL4*, *OsPAL6*, and *OsPAL7* were all upregulated DEGs in response to N-deficiency stress.

### 2.7. Modulation of Glutathione Metabolism in N-Deficiency Stress

Glutathione (GSH) plays a role in detoxification, antioxidant reactions, and redox homeostasis [65,66]. Although rice prefers ammonium as a N source, significant accumulation of GS2 in the roots and ammonium assimilation may cause subcellular condensation of ammonium, which can lead to ammonium toxicity and, ultimately, cell death, and suppressed growth and development [67,68]. Therefore, here, we found that GSH-involved detoxification was reasonably activated in both the transcriptome and the proteome after N-deficient treatment. In total, 20 DEGs (17 upregulated and 3 downregulated) and 12 DAPs (seven upregulated and five downregulated) were identified and listed (Supplementary Materials Table S9; Table 2 and Table 3).

Under N-deficiency stress, 11 glutathione S-transferases (GSTs) and 1 glutathione transferase (GT) were detected from the DEGs, and six GSTs and one GT were detected from the DAPs. Glutathione synthetase (GSS, B8BM87), the homolog of AtGSH2, was the most upregulated DAP from the glutathione pathway after 1/4N treatment. Notably, two DEGs and two DAPs related to ascorbate metabolism were all downregulated; of these, ascorbate peroxidase (*OsAPX3*, LOC_Os04g14680) is activated in heat shock and cadmium stress [69]. Ascorbate (ASA) and GSH have been reported to be antioxidants that scavenge reactive oxygen species. However, the chemical reaction of GSH with H_2_O_2_ is slow, and three peroxidases, namely, APXs, peroxiredoxins (PRXs), and GSTs, are known to link peroxide reduction to GSH oxidation [66,70,71,72]. Only one glutathione peroxidase (*OsGPX1*, LOC_Os04g46960) was moderately upregulated among the DEGs in the 1/4N treatment transcriptome [73,74]. Therefore, our results showed that GSH oxidation was linked to GST, rather than APX and PRX, was the preferred pathway in N-deficiency responses, all of which suggested that N starvation and ammonium toxicity can induce specific groups of GST pathway genes.

### 2.8. Transcription Factors (TFs) and Plant Hormones Involved in N-Deficiency Stress

TFs and plant hormones can regulate the expression of other genes and play a central role in regulating plant growth and adaptation to biotic and abiotic stresses, such as sensing nutrient availability [75]. Compared with 1N treatment, 1/4N treatment led to more upregulation of TFs. The relatively large TF families, including the WRKY, MYB, ERF, bHLH, and NAC families were found to correspond to N deficiency. The WRKY family—known to be involved in the regulation of plant root development [76]—included the most active TFs identified, and 33 of its members were found (including 31 upregulated and 2 downregulated). In total, 26 MYB TFs were detected after 1/4N treatment (19 upregulated and 7 downregulated), and, of 25 ERF family members detected, 23 were upregulated. The expression of 18 bHLH TFs was increased, but that of 6 bHLH TFs was suppressed. The number of the NAC TFs was 24 (21 upregulated and 3 downregulated) under N-deficient conditions (Supplementary Materials Figure S5). In general, most TFs were upregulated under N-deficient conditions (1/4N treatment) and unchanged or downregulated under N-sufficient conditions (1N treatment). These results indicated the presence of a very complex transcriptional regulation mechanism in the response of rice roots to low N supply.

**Figure 6:**
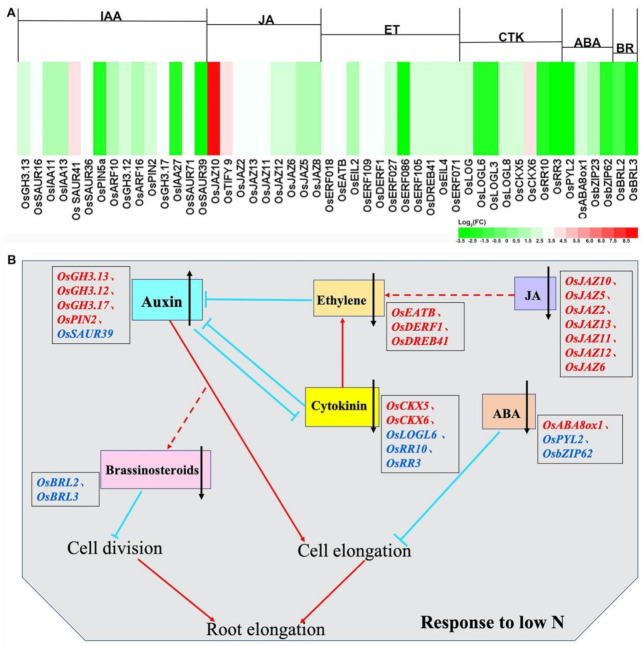
Expression profiles of genes associated with plant hormone signal transduction. (**A**) Heatmaps show the gene expression patterns related to IAA, JA, ET, CTK, ABA, and BR using log_2_fold change (FC) values; (**B**) genes involved in plant hormonal pathways to regulate root elongation after low N stress. Genes marked in red/blue represented upregulation and downregulation, respectively. IAA: indole-3-acetic acid, JA: jasmonic acid, ET: ethylene, CTK: cytokinin, ABA: abscisic acid, BR: brassinosteroids. FPKM: fragments per kilobase of transcript per million fragments mapped. Red arrows, blue blunted lines, and red dashed lines indicate positive, negative, and synergistic effects, respectively.

The results of the transcriptomic analysis based on KEGG pathways indicated that plant hormone signal transduction was significantly enriched under the lower N condition. Therefore, DEGs related to plant hormones, including indole-3-acetic acid (IAA), jasmonic acid (JA), ethylene (ET), cytokinin (CTK), abscisic acid (ABA), and brassinosteroids (BR), were visible on the heatmaps (Figure 6A). Under 1/4N treatment, the genes related to biosynthesis and transport in the IAA pathway, such as *OsGH3.13*, *OsGH3.12*, *OsGH3.17*, and *OsPIN2*, were upregulated, whereas *OsSAUR39*, the negative regulator of IAA synthesis and transport, was downregulated. These results suggested that enhanced IAA biosynthesis might stimulate root structure changes. In the CTK pathway, the expression of cytokinin dehydrogenases *OsCKX5* and *OsCKX6* was induced, whereas that of the cytokinin-activating enzyme *OsLOGL6* and the cytokinin-response regulators *OsRR10* and *OsRR3* was downregulated under N deficiency. The negative regulatory factors limiting ET synthesis, such as *OsEATB*, *OsDERF1*, and *OsDREB41*, were highly expressed under 1/4N treatment. In addition, the positive regulators of ABA, *OsPYL2* and *OsbZIP23*, were downregulated, but *OsABA8ox1* (ABA 8′-hydroxylase) was considerably upregulated under N-deficient conditions. BR receptor kinases *OsBRL2* and *OsBRL3* were also observed to be downregulated. These results were consistent with those of previous studies: IAA was a positive regulator of PR growth, whereas ET, CTK, ABA, and BR were negative regulators of primary root growth [47]. Additionally, *OsJAZ2*, *OsJAZ5*, *OsJAZ6*, *OsJAZ8*, *OsJAZ10*, *OsJAZ11*, *OsJAZ12*, and *OsJAZ13*—from the jasmonate ZIM-domain (JAZ) protein family, which is involved in JA signaling suppression [77,78]—were all upregulated after the 1/4N treatment. This result indicated that the aforementioned genes may also play a role in root morphological adaptation to the N-deficiency signal (Supplementary Materials Table S10; Figure 6B).

## 3. Discussion

### 3.1. Root Morphological Changes in N-Deficiency Stress

The root system architecture (RSA) is a highly modulated morphological system, affected by variations in the availability of nutrients [79,80,81], such as N [82]. Rice roots demonstrated a typical monocotyledon secondary homorhizic RSA; thus, PRs, crown roots (CRs), and LRs form in the 14-day-old rice seedlings [83,84,85,86,87]. In this study, LRs were almost inhibited in the high-NUE variety LY9348 (Figure 1B). This result is contradictory to that in *Arabidopsis*, where LRs are largely induced by N shortage [80,88,89,90,91,92]. Therefore, the absorption area increments on the RSA by LR initiation and elongation may not be the key mechanism underlying increased N absorption efficiency in LY9348, especially compared with the LRs in *Arabidopsis*. Because our study was performed in a hydroponic solution, LR induction by imbalanced distribution of N supply could not be analyzed in LY9348—and this could be a future research direction.

PR and LR elongation can be induced by a moderate level of N reduction, but inhibited by severe N limitation [79,84,89,91,92]. Here, LR elongation was not noted in LY9348; therefore, LY9348 may demonstrate a different LR modulation. PR elongation was observed in LY9348, and the longest PR was detected after 1/4N treatment in LY9348 (Figure 1A,B). Therefore, the threshold value for N shortage may be much lower in the high-NUE rice variety.

### 3.2. New GS Pathway for N Assimilation under N-Deficiency Stress

GS catalyzes NH_4_^+^ and glutamate (Glu) to form glutamine (Gln), which is the key reaction for inorganic N assimilation; however, the reproduction of Glu molecules for the sustainable GS reactions is supported by the GS/glutamine:2-oxoglutarate amidotransferase (GS/GOGAT) pathway—where one molecule of Gln and one molecule of 2-oxoglutaric acid (2-OG) are catalyzed to produce two molecules of Glu [12,13,17,93,94,95]. In this study, the chloroplast-expressed GS2 was found to be highly concentrated in the roots, thus increasing GS activity ectopically [38,39,40,41,66,96]. However, the key NADH-GOGAT1 protein in roots was not found to increase according to the considerable increments in GS2 enzymatic activity [16]. However, 2-OG production was noted to have increased significantly, based on the data profiling analysis (Supplementary Materials Table S2; Figure 2A). For example, enolase (B8AKN2, LOC_Os03g15950)—the enzyme responsible for the reversible conversion of D-2-phosphoglycerate (2PGA) and phosphoenolpyruvate (PEP) in glycolysis and gluconeogenesis [97]—was upregulated at 26 times higher in the 1/4N condition than in the 1N condition. In contrast, D-fructose-1,6-biphosphate 1-phosphohydrolase (FBPase, A7J2C3, LOC_Os05g36270)—which catalyzes hydrolysis of D-fructose-1,6-biphosphate (FDP) to D-fructose-6-phosphate (F6P) in gluconeogenesis [98]—was downregulated at 25 times lower in the 1/4N condition than in the 1N condition. These results suggested that glycolysis was stimulated to produce more FDP and PEP. Consequently, more PEP that entered into the TCA cycle led to the production of more 2-OG. Although no significant changes in OsNADH-GOGAT1 were observed, this enzyme was activated mainly in the roots, and it was detected at both the transcriptional and the translational levels. Besides, it was observed that relatively more Glu molecules were produced in the roots after 1/4N treatment (Figure 3D). Therefore, a sufficient number of Glu molecules were generated to support the upregulated GS2 enzymatic activity by other metabolic pathways than the known GOGAT cycle. For instance, the phenylalanine pathway and glucose/glycogenesis pathway were highlighted in the proteome profiling (Supplementary Materials Table S3). These observations supported the conclusion that secondary metabolites and glycolytic byproducts are generated in an active status.

The unexpected concentration of GS2 in the roots led to generation of more Gln molecules (Figure 3C) [14,99,100,101,102]. Considering that Gln is a signal molecule for N assimilation, the extra Gln molecules produced may thus be transported up to the shoot (Figure 3C) where it may interact with GOGAT to complete their metabolic reactions [13,103]. Because Gln can be recycled to produce more Glu by GOGAT in both the root and the shoot, increasing the protein level of each GOGAT enzyme (NADH-GOGAT1 in the root and NADH-GOGAT2/Fd-GOGAT in the shoot) is not necessary to save the energy for N searching. This is an excellent strategy that can be used for integrating the whole network into a N-usage-efficient mode. Interestingly, OsNADH-GOGAT2—expressed mainly in leaves—was noted to be upregulated in the transcriptome of the 1/4N-treated roots as a long-term response to the N-deficiency signals (Figure 4C). Therefore, the GS/GOGAT gene network must be actively regulated according to the variable N supply at multiple spatiotemporal levels in LY9348.

### 3.3. Glutamine as a Signal Molecule for N Assimilation and Transportation

During seed germination, the stored proteins are hydrolyzed rapidly to provide soluble amino acids; here, Gln is one of the major transported amino acids that is released [17,18,93,102,104]. The PR tip is sensitive to Glu; in *Arabidopsis*, Glu inhibits PR growth and stimulates LR outgrowth around the root tips of PR [80,105], which was inconsistent with our results. Glu is also the precursor for chlorophyll synthesis and is maintained relatively stable during the light/dark cycle, whereas the Gln concentration is observed to fluctuate dramatically [13]. Moreover, under the presence or absence of CO_2_, ammonium or nitrate induces N assimilation, which influences Glu and Gln concentrations differently in the leaves and the roots [13,106,107]. Therefore, Glu and Gln, either applied externally or generated internally, play complicated roles in N-related signal transduction and metabolic processes. In particular, the endogenous levels of Gln are considered to be markers of the cellular N status.

In this study, although N sources were supplied both in ammonium and nitrate forms with a 1:1 ratio, the unexpected concentration of GS2 in the roots suggested that LY9348 prefers ammonium over nitrate, even in the N-deficient conditions (Figure 3A). Sonoda et al. [100] showed that ammonium induced significant increases in Gln levels within 4 h of its application on the rice roots. A considerable number of Gln molecules were produced, as expected, in LY9348 roots after 1/4N treatment (Figure 3C). However, the absence of increments in GOGAT enzymatic activity in the roots, as well as with the promotion of PR elongation, together suggest that the generated Gln must be transported from the roots to the shoots very rapidly, so as to reduce Gln accumulation in the roots. Additionally, the upregulation of Glu content after 1/4N treatment suggested that the local GOGAT enzymes in the shoot and root may be involved in Gln molecule recycling, which is an efficient strategy to balance Gln/Glu biochemically (Figure 3D).

### 3.4. Relatively Normal Shoot Growth in N-Deficiency Stress

In this study, when PR elongation was at its maximum in LY9348 under the 1/4N condition, the photosynthesis, chlorophyll content, biomass accumulation, and Gln and Glu levels in its shoots were at normal levels—or they showed increased levels compared with the other N conditions (Supplementary Materials Figure S1B; Figure 3C–F). This suggested that the high-NUE variety adopts a different mechanism to rescue the plant when N supply is limited; this mechanism is relatively more active and efficient, improving its inner N recycling and usage. All regulatory DEGs were summarized and presented using Mapman (Supplementary Materials Figure S6); here, multilevel regulation, as well as balance between signaling transduction, cell wall metabolism, TFs (Supplementary Materials Figure S5), plant hormones (Figure 6), and secondary metabolites could also be noted [42,80,102,108,109,110,111,112,113,114,115].

Previous studies have shown that the elaborate hormone signaling crosstalk networks and regulation function of TFs make them ideal candidates for mediating root morphological changes in N-deficiency stress [116,117]. Several TFs, including MYB, WRKY, NAC, bHLH, bZIP, and MADS-box, participate in the plant development regulation in a N-dependent manner [118,119]. For example, *OsMADS57* regulates long-distance nitrate transport and root elongation via *OsNRT2.3a* [120], and *NAC42*-activated nitrate transporter confers high NUE in rice [36]. Notably, CTK levels increased in maize roots, potentially antagonizing IAA action, as well as promoting ET production and, thus, inhibiting root elongation under high N supply [43]. This is consistent with our results (Figure 6). Recently, ET was reported to participate in low NH_4_^+^-induced root hair elongation in Arabidopsis seedlings [121]. Additionally, IAA, CTK, and ABA have been reported to play critical roles in the plant response to fluctuating availability of useable N [108,122]. Sun et al. revealed the involvement of JA signaling and the transcription factor OsJAZ9 in reprogramming of N uptake and metabolism [123]. In addition, *OsERF2*, involved in ET signaling, was shown to regulate the accumulation of sucrose and UDPG, exerting a significant regulatory role in rice root growth [124]. Here, hormone-related transcripts, in particular, those pertaining to the JA signaling pathway, were upregulated significantly under N starvation (Figure 6). All these indicate significant roles of TFs and plant hormone signaling in the low-N response in the rice root.

Due to GS2 accumulation, lignin synthesis involved in the phenylpropanoid biosynthesis pathway was highly upregulated, resulting in thickened cell wall regulation to shape PR elongation. Moreover, secondary metabolites are positively regulated to support the fast changes caused by the aforementioned biochemical reactions [56,125,126]. In the redox state, peroxidases (Supplementary Materials Tables S8 and S9; Table 2 and Table 3), especially GSTs, are activated to resolve ammonium toxicity [57,65,68,72]. Therefore, normal shoot development and active photosynthesis are maintained to provide sufficient energy for prolonged RSA modifications for N searching in the roots, and to guarantee a larger chance of survival under N-deficiency stress.

### 3.5. Prolonged N-Deficiency Responses

Internal N is stored in the endosperm of the seeds, and its influences are largely reduced through longer, real N starvation stress. Therefore, after a N-deficiency stress, proteome profiling demonstrates the first-order response at the protein function level, whereas transcriptome profiling indicates the second-order response at the transcription level; this is the reason for the low correlation between the transcriptome and proteome datasets.

Notably, the unexpected concentration of GS2 in LY9348 roots may stimulate ammonium absorption, Gln production/transportation, and GOGAT-linked N:C homeostasis, which was sufficient to keep a high metabolite level and ensured high energy production and shoot and root biomass increase [13,15,93]. This was a strategy considerably successful as the first-order N-deficiency response. Nevertheless, in addition to ammonium transporters (*AMT1.2*, *AMT1.3*, and putative *AMT3.3*), many nitrate transporters (e.g., *NRT2.1*, *NRT2.2*, *NRT2.3a*, and *NRT2.4*), partner protein for high-affinity nitrate transport (*NAR2.1*), and nitrate reductase (*NR2*) were upregulated at the transcriptional level—suggesting that nitrate uptake is part of the second-order N-deficiency response [4,14,42,80,99,101,102,127,128,129,130,131]. Moreover, DEGs, such as *GS1;1*, *GS1;2*, *GS1;3*, and *NADH-GOGAT2*, were also upregulated, suggesting that another GS pathway was stimulated under prolonged N deficiency [12,13,16,17] (Figure 4C). Therefore, further analysis using different N sources, shorter and longer treatment durations, and older developmental stages for LY9348 is warranted to clearly elucidate the high-NUE pathways in rice and shed light on methods for increasing N usage efficiency in agricultural crops.

## 4. Materials and Methods

### 4.1. Plants and Materials

LY9348 and 9311 seeds were soaked in water at 28 °C for 24 h to stimulate germination. Healthy, uniformly germinated seeds were picked and transferred individually into each cell of PhytoTC seed germination pouches (PhenoTrait Technology, Beijing, China). Next, 30 mL of hydroponic nutrient solution was added into each pouch to start a hydroponic culture. The hydroponic solution was prepared according to International Rice Research Institute Protocol: 2.9 mM NH_4_NO_3_, 1 mM KCl, 1 mM CaCl_2_, 1 mM KSO_4_, 0.32 mM NaH_2_PO_4_•2H_2_O, 1.7 mM MgSO_4_•7H_2_O, 9.1 × 10^−3^ mM MnCl_2_•4H_2_O, 5.2 × 10^−4^ mM (NH_4_)_6_MoO_2_•4H_2_O, 1.8 × 10^−2^ mM H_3_BO_3_, 1.5 × 10^−4^ mM ZnSO_4_•7H_2_O, 1.6 × 10^−4^ mM CuSO_4_•5H_2_O, 3.6 × 10^−2^ mM FeCl_3_•6H_2_O, and citric acid, and pH 5.5–6.0. Here, the amounts of NH_4_NO_3_ were manipulated to obtain six different concentrations in the culture solution: 0N, 0 mM NH_4_NO_3_; 1/4N (25% N), 0.725 mM NH_4_NO_3_; 1/2N (50% N), 1.45 mM NH_4_NO_3_; 1N (100% N), 2.9 mM NH_4_NO_3_ (as in the original recipe); 2N (200% N), 5.8 mM NH_4_NO_3_; and 4N (400% N), 11.6 mM NH_4_NO_3_. All plants were grown in a growth chamber (E-36L, Percival, IA, USA) with 12-h light (30 °C)/12-h dark (28 °C) photoperiod cycle, 200 µM/m^2^/s photon density, and 80% humidity [24]. The nutrient solutions were refreshed every 3 days, and the treatments were performed continuously for 2 weeks. All shoots and roots were harvested and separated, quickly frozen in liquid nitrogen, and stored at −80 °C for analysis. For each N treatment, 20 individual plants were included, with three biological repeats being included per treatment.

### 4.2. Phenotypic Analysis

During hydroponic culture time, the root length of six individual plants was measured and recorded on the 1st, 2nd, 5th, 7th, 9th, 12th, and 14th day after germination for each treatment. The average value for six measured individuals was recorded as the root length of the treatment for that day, and each treatment was repeated three times. When 14-day treatments were completed, fresh plants were harvested; the shoots and roots were separated to measure the FW. The fresh shoots and roots were then baked in the oven at 70 °C until they were dried out, and the DW of each part was measured. For each weight measurement, six individual plants were included, and three biological replicates were used for each treatment.

### 4.3. RNA Extraction and Sequencing

Sixty individual plants of LY9348 were harvested after 14 days of treatment in 1N and 1/4N hydroponic solutions. The roots of these plants were isolated, mixed, and ground with liquid nitrogen. Total RNA was extracted using the TRIzol (Invitrogen, Carlsbad, CA, USA), according to the manufacturer’s instructions. The total RNA quality was verified on a 2100 Bioanalyzer (Agilent Technologies, Santa Clara, CA, USA) and quantified using a Nanodrop2000C Spectrophotometer (Thermo Fisher Scientific, Waltham, MA, USA). The exact standard of plant samples for transcriptome sequencing is as follows: RNA integrity number (RIN) ≥ 6.5, 28S:18S ≥ 1, OD_260/280_ = 1.8–2.2, and OD_260/230_ ≥ 2.0.

For sequencing library preparation, we used NEBNext Ultra RNA Library Prep Kit for Illumina (NEB, Ipswich, MA, USA), according to the manufacturer’s instructions. In brief, mRNA was purified from a total of 3 μg of RNA per sample as input material by using poly-T oligo-attached magnetic beads. Next, fragmentation was performed using divalent cations under high temperature in NEBNext First Strand Synthesis Reaction Buffer. First-strand cDNA was synthesized using a random hexamer primer and M-MuLV reverse transcriptase. Second-strand cDNA synthesis was subsequently performed using DNA polymerase I and RNase H. The end of double-stranded cDNA (dsDNA) was repaired via exonuclease and polymerase activities, and then A (dA-tailing) was added to 3′ ends of dsDNA. NEBNext adaptor with a hairpin loop structure was added to each sample. Subsequently, the fragments were purified on an AMPure XP system (Beckman Coulter, Beverly, MA, USA) to select cDNA fragments of preferentially 150–200 bp in length. Then, we exposed size-selected adaptor-ligated cDNA to a temperature of 37 °C for 15 min, followed by 95 °C for 5 min, and 3 μL of USER Enzyme (NEB, Ipswich, MA, USA) before PCR. Next, PCR was performed with Universal PCR primers, Phusion High-Fidelity DNA polymerase, and Index (X) Primer (NEB, Ipswich, MA, USA). Finally, PCR products were purified using the AMPure XP system (Beckman Coulter, Fullerton, CA, USA), and library quality was evaluated on the Agilent Bioanalyzer 2100 system (Agilent Technologies, Santa Clara, CA, USA).

Then the clustering of the index-coded samples was carried out on a cBot Cluster Generation System using TruSeq PE Cluster Kit v3-cBot-HS (Illumina, San Diego, CA, USA), according to the manufacturer’s instructions. The library preparations were sequenced on an Illumina Hiseq 2500 platform (Illumina) and 150-bp paired-end reads were produced after cluster generation.

For quality control, raw data (i.e., raw reads) of fastq format were first filtered through in-house perl scripts, and clean data (i.e., clean reads) were obtained by removing raw read-containing adapter, poly-N, and low-quality reads. Moreover, Q30, GC content, and sequence duplication level of clean data were calculated. The clean reads were aligned to the Nipponbare reference genome (http://rice.plantbiology.msu.edu, accessed on 25 May 2020) using HISAT2 v2.0.5, and the mapped reads were assembled and quantified using StringTie.

Finally, the all gene expression level of two samples were assessed as FPKM (fragments per kilobase of transcript per million fragments mapped) by using FeatureCounts v1.5.0-p3 as follows:

$$\mathrm{FPKM}=\frac{\mathrm{Number}\;\mathrm{of}\;\mathrm{cDNA}\;\mathrm{fragments}}{\mathrm{Number}\;\mathrm{of}\;\mathrm{mapped}\;\mathrm{fragments}\;\left(\mathrm{in}\;\mathrm{million}\right)\;\times \;\mathrm{Transcript}\;\mathrm{length}\;(\mathrm{in}\;\mathrm{kb})}$$

The DEGs between two samples were performed using the DESeq2 R package. The Benjamini–Hochberg correction method was then applied to correct for the significant *p* value obtained from the original hypothesis test, and FDR was used as the key indicator for screening DEGs. Then, the significant DEGs were defined using |FC| ≥ 2.0, FDR < 0.01 and adjusted *p* < 0.05. Each sample containing 60 mixed individuals was collected for RNA-Seq, and two replicates of each sample were included.

### 4.4. qRT-PCR Validation

Total RNA was extracted from 2-week-old seedling roots of LY9348 using the TRIzol reagent (Invitrogen), precipitated using isopropyl alcohol, and dissolved in DEPC-treated water for analysis. The RNA concentration was determined on Nanodrop2000C Spectrophotometer (Thermo Fisher Scientific). Then, 1 μg of RNA was reverse-transcribed for first-strand cDNA synthesis using HIScriptIII 1st Strand cDNA Synthesis Kit (+gDNA wiper; Vazyme, China), according to the manufacturer’s instructions. Specific primers were designed using the Primer Premier 5.0 (Supplementary Materials Table S11). *OsActin* (LOC_Os03g50885) was used as the housekeeping gene to normalize cDNAs. qRT-PCR was performed using Hieff qPCR SYBR Green Master Mix (YESEN, Shanghai, China) on a LightCycler 480 system (Roche, Mannheim, Germany). The reaction program was set as follows: 95 °C for 10 s, followed by 40 cycles of 95 °C for 15 s, and, finally, 60 °C for 30 s; the PCR product was then quantified using the 2^−ΔΔCt^ method [132]. Three biological replicates were included for this experiment, and each sample included 20 mixed individuals.

### 4.5. Protein Extraction, Digestion, and Identification by Using Label-Free Technology

The plant materials, including 60 mixed individuals, used for label-free proteome quantification were the same as those used for RNA-Seq. The protein extraction was performed through Tris-acetone extraction, as described previously [133]. For protein lysis, a lysis buffer (7 M urea; 2 M thiourea; 4% SDS; 40 mM Tris-HCl, pH 8.5; 1 mM PMSF; 2 mM EDTA) was added to the samples and mixed well. This was followed by incubation on ice for 5 min and ultrasonication in an ice bath for 15 min after the addition of DTT to reach a final concentration of l0 mM. After this, the solution was centrifuged at 4 °C for 20 min at 13,000× *g*, the supernatant was transferred to a new centrifuge tube, followed by the addition and mixing of a 4X volume of cold acetone. Next, DTT was added to a final concentration of 30 mM. This was followed by mixing and incubation at −20 °C overnight for precipitation. Next, the protein precipitates were collected through centrifugation at 4 °C and 13,000× *g* for 20 min, and then air dried. Subsequently, 8 M urea/100 mM TEAB (pH 8.0) was added to dissolve the protein and DTT was added to achieve a final concentration of 10 mM. Reduction reaction was initiated in a 56 °C water bath for 30 min. Subsequently, IAM was added to the final concentration of 55 mM, and the alkylation reaction was performed at room temperature in the dark for 30 min. Protein concentration was determined using a protein assay kit based on the Bradford method from Bio-Rad (Hercules, CA, USA) with bovine serum albumin as the standard. Equal amounts of proteins (100 μg) from each sample were used for digestion with trypsin (Promega, Madison, WI, USA). The protein solution was diluted five times with 100 mM TEAB, and trypsin was added to reach a pancreatin-to-protein mass ratio of 1:50. Next, enzymolysis was performed at 37 °C overnight. Desalination of enzymolyzed peptides was implemented on C18 columns (Phenomenex, Torrance, CA, USA) with vacuum drying (IPC250-3, LEADER, Shanghai, China).

The TripleTOF 5600+Liquid Chromatography–Mass Spectrometry System (AB SCIEX, Framingham, MA, USA) was employed for mass spectrometry data collection. Polypeptide samples were dissolved in 2% acetonitrile/0.1% formic acid and analyzed on a mass spectrometer, coupled with an Eksigent nanoLC 400 System (AB SCIEX, Framingham, MA, USA). The polypeptide solution was added to a C18 capture column (5 μm, 100 μm × 20 mm; Phenomenex, Torrance, CA, USA), and gradient elution was performed on a C18 analysis column (3 μm, 75 μm × 150 mm, Phenomenex, Torrance, CA, USA) with a 90-min time gradient and 300-nL/min flow rate. Two mobile phases were used: buffer A, 2% acetonitrile + 0.1% formic acid + 98% H_2_O, and buffer B, 98% acetonitrile + 0.1% formic acid + 2% H_2_O. For information-dependent collection (IDA), the first-order mass spectrometry (MS1) was scanned with an ion accumulation time of 250 ms and the second-order mass spectrometry (MS2) of 40 precursor ions was collected using an ion accumulation time of 50 ms. The MS1 spectrum was accumulated in the range of 350–1500 *m*/*z*, and the MS2 spectrum was collected in the range of 100–1500 *m*/*z*.

The database was downloaded from the Uniprot Database *Oryza sativa* protein sequence, which contains 23,896 protein sequences (https://www.uniprot.org, accessed on 13 June 2020). The filter criterion for identification was that the reliability of peptide segment is >95% and the identified protein contains at least one unique peptide segment. FDR was calculated using the Proteomics System Performance Evaluation Pipeline, and only unique peptides with global FDR values <1% were considered to indicate significant data. All the identified proteins were quantitatively analyzed using Skyline v3.6. Here, the screening criteria for significant DAPs were different multiples of |FC| ≥ 2. The hypergeometric test was applied to calculate the *p* value, with *p* < 0.05 as the threshold.

### 4.6. Protein and RNA Correlation Analysis

Correlation analysis was performed from the expression and functional enrichment aspects between transcriptome and proteome. According to their corresponding numbers on NCBI (https://www.ncbi.nlm.nih.gov, accessed on 13 August 2020) and Uniprot (https://www.uniprot.org, accessed on 13 August 2020), mRNAs and proteins were respectively integrated for analysis and eventually associated with their source gene. The expression rates based on log_2_FC of DEGs and DAPs were defined as the parameters of correlation analysis. The DEGs and DAPs were screened using |FC| ≥ 2 and |FC| ≥ 1.5, respectively, and a *p* value of < 0.05 was also used as a criterion. Comparative correlation analysis of different KEGG pathways in the two groups was also performed, and significant KEGG pathway enrichment was assessed using the Pearson analysis, with *p* < 0.05 indicating significance.

### 4.7. GO, KEGG Enrichment Analysis, and Functional Annotation of DEGs and DAPs

All the selected DEGs and DAPs were mapped to each term in the GO database (http://www.geneontology.org, accessed on 20 August 2020) and the number of genes and proteins in each term were counted. GO enrichment analysis was implemented by the GOseq R package based on Wallenius noncentral hypergeometric distribution, and GO terms with a corrected *p* value of < 0.05 were considered significantly enriched by DEGs or DAPs. The KEGG pathway enrichment analysis was conducted using the KOBAS v3.0 website (http://kobas.cbi.pku.edu.cn/kobas3, accessed on 22 August 2020). We identified significantly enriched metabolic pathways by using the hypergeometric test, and the significantly enriched KEGG pathways with a corrected *p* value of< 0.05 were considered.

All DEG and DAP functions were annotated using the National Rice Data Center (http://www.ricedata.cn, accessed on 27 August 2020) and Uniprot (https://www.uniprot.org, accessed on 27 August 2020) websites, respectively. The TFs of DEGs were classified using the PlantTFDB website (http://planttfdb.gao-lab.org, accessed on 28 August 2020).

### 4.8. GS Enzymatic Activity Assays

GS enzymatic activity was analyzed by the Glutamine Synthetase Assay Kit (JianCheng Bioengineering Institute, Nanjing, China). Plant extracts were prepared by grinding 0.1 g of fresh leaves or 0.1 g of roots from 2-week-old seedlings and mixed with 1 mL of extraction buffer (tissue weight (g): extract volume (mL) = 1:10). Extraction mixture was homogenized at 4 °C in an ice-water bath, and then centrifuged at 4000× *g* for 10 min at 4 °C. Next, the supernatant was collected on ice, followed by GS activity assay. In brief, 175 μL of the extracted supernatant was mixed well with 825 μL of the reaction mixture in a quartz cuvette, which was then inserted into a spectrophotometer (V-5100B, Shanghai METASH Instrument Co., Ltd., Shanghai, China). Here, we recorded the GS enzymatic activity (μmol/h/g) spectrophotometrically as the optical density (OD) value of the reaction complex formed at 540 nm according to the standard curve:

$$\frac{\Delta A\;-\;0.0008}{0.8348}\;\times \;\mathrm{total}\;\mathrm{volume}\;\mathrm{of}\;\mathrm{reaction}\;\mathrm{solution}\;÷\;\left(\mathrm{sample}\;\mathrm{weight}\;\times \;\mathrm{sampling}\;\mathrm{volume}\;÷\;\mathrm{extract}\;\mathrm{volume}\right)\;÷\;\mathrm{reaction}\;\mathrm{time}$$

(The formula of standard curve:$\;y=0.8348x+0.0008,\;R^{2}=0.9999)$.

For each treatment, at least five mixed individuals were included, and three biological replicates of each treatment were included in the measurements.

### 4.9. Determination of Glu and Gln Contents

The Glu content was analyzed using a Glu Content Assay Kit (Beijing Boxbio Science & Technology Co., Ltd., Beijing, China). Here, the plant extract preparation process was the same as that used for GS activity assay. The plant extract was then centrifuged at 10,000× *g* for 10 min at room temperature. Next, the supernatant was collected for Glu content assay. In brief, 40 μL of the supernatant was mixed well with 170 μL of the reaction mixture in a 96-well UV plate. Then we recorded the absorbance of each well at 340 nm on an enzyme-labeled instrument (SpectraMax**^®^** iD5, Molecular Devices, Silicon Valley, CA, USA), respectively. Glu content (μmol/g) was calculated using the following equation:

$$\frac{\Delta A\;-\;0.0362}{0.3953}\;\times \;\mathrm{sampling}\;\mathrm{volume}\;\times \;\mathrm{extract}\;\mathrm{volume}\;÷\left(\mathrm{sample}\;\mathrm{weight}\;\times \mathrm{sampling}\;\mathrm{volume}\right)$$

The Gln content was quantified using a Gln Content Assay Kit (Suzhou Grace Biotechnology Co., Ltd., Suzhou, China). Plant sample (0.1 g) was mixed with extraction buffer and ground well. This was followed by centrifugation at 12,000× *g* for 10 min. Next, the supernatant was collected for determining Gln content. Briefly, 20 μL of the supernatant was mixed thoroughly with 180 μL of the reaction mixture in a 96-well plate, followed by incubation at 30 °C for 20 min in a dark place. Next, the absorbance of each well at 450 nm on an enzyme-labeled instrument (SpectraMax^®^ iD5, Molecular Devices, Silicon Valley, CA, USA) was recorded, and the following equation was used to calculate Gln content (μmol/g):

$$\frac{\Delta A+0.0012}{0.0824}\;÷\;\left(\mathrm{sample}\;\mathrm{weight}\;\times \;\mathrm{sampling}\;\mathrm{volume}\;÷\;\mathrm{extract}\;\mathrm{volume}\right)\times 10^{-3}$$

(The formula of standard curve:$\;y=0.0824x\;-\;0.0012{,\;R}^{2}\;=0.997)$.

For each treatment, five individual samples were mixed, and three biological replicates of each treatment were included.

### 4.10. Determination of SPAD and Phi2 Values

After the 2-week 1N or 1/4N treatment was completed, the leaf chlorophyll content, denoted by the SPAD value, was measured on a SPAD-502 Plus chlorophyll meter (Spectrum Technologies, Plainfield, IL, USA). Moreover, the top fully spread leaf from each plant was picked to measure. Because the SPAD meter is a nondestructive equipment, each picked leaf was measured repeatedly three times. Then, the average of these measurements was taken as the value for the plant, and the average value of at least five individual plants was recorded as the final SPAD value for each treatment. Photosynthetic rate, presented as Phi2 (optical energy coefficient captured by photosystem II), was measured by MultispeQ (PhenoTrait Technology, Beijing, China). Then, the leaf selected for this and SPAD measurement was the same for each plant. Since MultispeQ is also a nondestructive equipment, each picked leaf was measured repeatedly three times and the average of these measurements was taken as the Phi2 value of the plant, and the average value of at least five individual plants was recorded as the final Phi2 value for each treatment. For each treatment, three biological replicates were included.

### 4.11. Statistical Analysis

All data were subjected to one-way analysis of variance (ANOVA), and significant differences (*p* < 0.05) between treatments were indicated by different letters according to Tukey’s HSD test. Data were shown as the means ± SE (*n* = 3), and the biological replicates were represented by *n*. Graphpad Prism version 5.0 (GraphPad software, San Diego, CA, USA) and SPSS software version 25 (IBM Corporation, New York, NY, USA) was used for graphic illustrations and statistics calculations.

## 5. Conclusions

Our current results revealed a global view of the morphological changes in LY9348 roots under N-deficient conditions. Accordingly, an alternative molecular network of chloroplastic GS2 unexpectedly involved in N-foraging regulation for PR elongation and shoot growth in LY9348 was obtained on the basis of the transcriptomics and proteomics data. The related genes or proteins involved in phenylpropanoid biosynthesis and glutathione metabolism pathways were noted to have significantly changed under N-deficiency stress. Additionally, plant hormones and TFs were noted to play vital roles in the adaptation of LY9348 to low N conditions, as well as in the regulation of its root architecture.

## Figures and Tables

**Table 1:** The part of gene list for correlation analysis of transcriptome and proteome.

Class	Protein ID	MSU_Locus	Gene Annotation
Com_down	sp|Q6K4E7|APM1D	LOC_Os09g19800	aminopeptidase
	sp|Q7XUG1|MASY	LOC_Os04g40990	glyoxysomal
Com_up	sp|Q5WA72|PDI15	LOC_Os06g06790	OsPDIL1-5 protein disulfide isomerase
	sp|Q7PC80|AB34G	LOC_Os01g42350	pleiotropic drug resistance protein
P_up_T_down	sp|Q6Z9A3|GMPP3	LOC_Os08g13930	GDP-D-mannose pyrophosphorylase
	sp|B8BH95|ITPA	LOC_Os10g31940	inosine triphosphate pyrophosphatase
P_down_T_up	sp|Q06398|GSTU6	LOC_Os01g37750	glutathione S-transferase
	sp|Q10LX4|CML27	LOC_Os03g21380	OsCML27
	sp|Q10S72|4CLL4	LOC_Os03g04000	AMP-binding domain containing protein
	sp|O04985|HBL2	LOC_Os03g12510	non-symbiotic hemoglobin 2
	sp|Q42997|NIR	LOC_Os02g52730	ferredoxin--nitrite reductase

Com_down: downregulated in both data profiling; Com_up: upregulated in both data profiling; P_up_T_down: upregulated in proteome but downregulated in transcriptome; P_down_T_up: downregulated in proteome but upregulated in transcriptome.

**Table 2:** The DAPs involved in phenylpropanoid biosynthesis and glutathione metabolism pathway in 1/4N_1N comparison.

Protein ID	MSU_Locus	Annotation	FC(1/4N/1N)
**Phenylpropanoid Biosynthesis (ko00940)**			
upregulated			
A2Y626	LOC_Os05g41440	cytochrome P450 98A1	3.76
A2ZCW8	LOC_Os11g12760	O-methyltransferase ZRP4	3.53
B8AND5	LOC_Os03g05780	4-coumarate--CoA ligase-like 7	2.42
Q6ZHS4	LOC_Os02g09490	cinnamyl alcohol dehydrogenase	3.37
A0A0N7KK39	LOC_Os05g04470	peroxidase precursor	4.44
A2YHC0	LOC_Os07g01420	peroxidase 1 precursor	2.66
downregulated			
A2XZ41	LOC_Os04g58710	peroxisomal-coenzyme A synthetase	0.10
B8ARF5	LOC_Os04g57850	AMP-binding protein gene	0.11
B8AMG2	LOC_Os03g19250	AMP-binding protein	0.25
Q10S72	LOC_Os03g04000	4-coumarate--CoA ligase-like 4	0.31
A2XB41	LOC_Os02g57480	anthocyanin 5-aromatic acyltransferase	0.49
A2Y375	LOC_Os05g25640	trans-cinnamate 4-monooxygenase	0.31
Q60DX8	LOC_Os05g30350	Os5bglu22—beta-glucosidase homologue	0.42
B8AV76	LOC_Os04g54810	beta-D-xylosidase	0.43
B8B5W7	LOC_Os07g48010	peroxidase 2 precursor	0.26
A2WZD6	LOC_Os01g73170	peroxidase 12 precursor	0.42
P0C5D0	LOC_Os07g44440	peroxiredoxin	0.47
**Glutathione Metabolism (ko00480)**			
upregulated			
B8BM87	LOC_Os12g34380	glutathione synthetase	12.30
A2YL42	LOC_Os07g28480	glutathione S-transferase	4.71
A2Z9K4	LOC_Os10g38580	glutathione S-transferase GSTU6	2.94
B8BI29	LOC_Os10g39740	glutathione S-transferase	2.42
A6N0E3	LOC_Os01g27390	glutathione transferase	2.24
A1XBB7	LOC_Os03g17470	IN2-1 protein	2.17
A2Z263	LOC_Os09g29200	glutathione S-transferase	2.03
downregulated			
A0A0P0UYM7	LOC_Os01g05820	gamma-glutamyltranspeptidase 1 precursor	0.23
Q06398	LOC_Os10g38740	glutathione S-transferase	0.12
Q65XA0	LOC_Os05g02530	cytosolic dehydroascorbate reductase	0.30
A2XC68	LOC_Os03g04250	glutathione S-transferase 6	0.46
Q7XJ02	LOC_Os04g35520	probable L-ascorbate peroxidase 7	0.42

**Table 3:** The candidate DEGs involved in phenylpropanoid biosynthesis and glutathione metabolism pathway in 1/4N_1N comparison.

Gene Locus	FDR	log_2_FC	Annotation
**Phenylpropanoid biosynthesis (ko00940)**			
upregulated			
LOC_Os06g29470	0	5.99	peroxidase 1-like
LOC_Os02g56700	0	4.45	cinnamoyl-CoA reductase 1
LOC_Os08g34790	0	2.92	Os4CL5
LOC_Os06g46799	0	2.90	peroxidase 3
LOC_Os03g25360	4.53 × 10^−12^	2.88	peroxidase 2
LOC_Os04g39880	4.51 × 10^−12^	2.35	Os4BGlu12
LOC_Os02g41670	2.91 × 10^−10^	2.18	OsPAL3
LOC_Os05g35290	1.14 × 10^−5^	1.65	OsPAL7
LOC_Os03g11420	1.48 × 10^−4^	1.53	Os3BGlu6
LOC_Os01g40860	1.04 × 10^−4^	1.52	OsALDH2C4
LOC_Os08g14760	7.57 × 10^−5^	1.52	Os4CL1
LOC_Os06g44620	9.84 × 10^−5^	1.50	Os4CL4
LOC_Os04g43800	8.16 × 10^−4^	1.35	OsPAL6
LOC_Os02g41680	1.88 × 10^−3^	1.34	OsPAL4
downregulated			
LOC_Os08g02110	3.72 × 10^−10^	−2.18	peroxidase 47
LOC_Os07g02440	4.12 × 10^−11^	−2.29	peroxidase 50
**Glutathione metabolism (ko00480)**			
upregulated			
LOC_Os10g38740	2.44 × 10^−15^	2.79	glutathione S-transferase GSTU6
LOC_Os03g44170	5.34 × 10^−12^	2.69	probable glutathione S-transferase GSTU1
LOC_Os09g20220	3.71 × 10^−11^	2.30	probable glutathione S-transferase GSTU1
LOC_Os04g46960	1.55 × 10^−8^	2.00	OsGPX1
LOC_Os01g72160	6.23 × 10^−7^	1.90	probable glutathione S-transferase
LOC_Os04g38450	8.48 × 10^−7^	1.82	gamma-glutamyltranspeptidase 1 precursor
LOC_Os10g38580	3.43 × 10^−6^	1.77	probable glutathione S-transferase GSTU6
LOC_Os10g38640	1.33 × 10^−6^	1.76	putative glutathione S-transferase
LOC_Os03g57200	1.07 × 10^−6^	1.76	glutathione S-transferase GSTU1
LOC_Os10g38600	2.61 × 10^−5^	1.57	putative glutathione S-transferase
LOC_Os01g70770	4.75 × 10^−4^	1.42	glutathione S-transferase 3
LOC_Os10g38314	7.88 × 10^−4^	1.37	glutathione S-transferase
LOC_Os09g29200	8.16 × 10^−4^	1.36	glutathione transferase GST23
LOC_Os11g29400	2.54 × 10^−3^	1.30	6-phosphogluconate dehydrogenase
LOC_Os10g38189	3.78 × 10^−3^	1.25	glutathione S-transferase
downregulated			
LOC_Os04g14680	1.61 × 10^−3^	−1.41	OsAPx3
LOC_Os03g29950	1.50 × 10^−3^	−2.08	glucose-6-phosphate 1-dehydrogenase
LOC_Os04g51300	4.07 × 10^−4^	−2.40	peroxidase precursor

## Data Availability

The raw data collected from RNA-Seq was availability in National Center for Biotechnology Information (NCBI) Sequence Read Archive (SRA) under accession number: PRJNA730211 (https://dataview.ncbi.nlm.nih.gov/object/PRJNA730211?reviewer=fn5ahp943h3r7r2qgskurhu3u5). The raw data collected from Proteome was deposited to integrated proteome resources (iProX) under the accession code: IPX0003070002 (https://www.iprox.org/page/DDV019.html?subProjectId=IPX0003070002&type=1).

## Supplementary Materials

The following are available online at https://www.mdpi.com/article/10.3390/ijms22147674/s1, Figure S1: Fresh weight (g) of the LY9348 in the roots (A) and in the shoots (B); Figure S2: Correlation analysis between qRT-PCR gene expression level and corresponding RNA-Seq data; Figure S3: Correlation diagram of expression ratio between transcriptome and proteome; Figure S4: Comparative analysis of GO annotation between DEGs and DAPs; Figure S5: The transcription factors (TFs) classification of DEGs analyzed through PlantTFDB website; Figure S6: The model of the regulation mechanism of rice roots in response to N starvation involves numerous pathways by MapMan software. Table S1: Statistics and details of DAPs in this study; Table S2: The candidate known function DAPs of interest for cluster analysis; Table S3: KEGG pathways associated with the number of up-regulated and down-regulated DAPs; Table S4: Statistics and details of DEGs in this study; Table S5: Significantly enriched KEGG pathways associated with the number of DEGs; Table S6: A list of the selected DEGs for qRT-PCR validation; Table S7: The correlation analysis between proteome and transcriptome; Table S8: The DEGs and DAPs involved in phenylpropanoid biosynthesis pathway in 1/4N_1N comparison; Table S9: The DEGs and DAPs involved in glutathione metabolism pathway in 1/4N_1N comparison; Table S10: The DEGs involved in plant hormone signal transduction in 1/4N_1N comparison; Table S11: Primers of mRNAs used in this study.
