# Supplementary material for: Integrative Transcriptomic and Proteomic Analysis Reveals an Alternative Molecular Network of Glutamine Synthetase 2 Corresponding to Nitrogen Deficiency in Rice (Oryza sativa L.)
Source: Int J Mol Sci. 2021 Jul 18;22(14):7674. doi: 10.3390/ijms22147674 (PMC8304609; doi:10.3390/ijms22147674)
Supplement: Supplementary file 1 [file ijms-22-07674-s001.zip › ijms-1238115-supplementary/Supplementary files/Supplementary Figure .pdf]

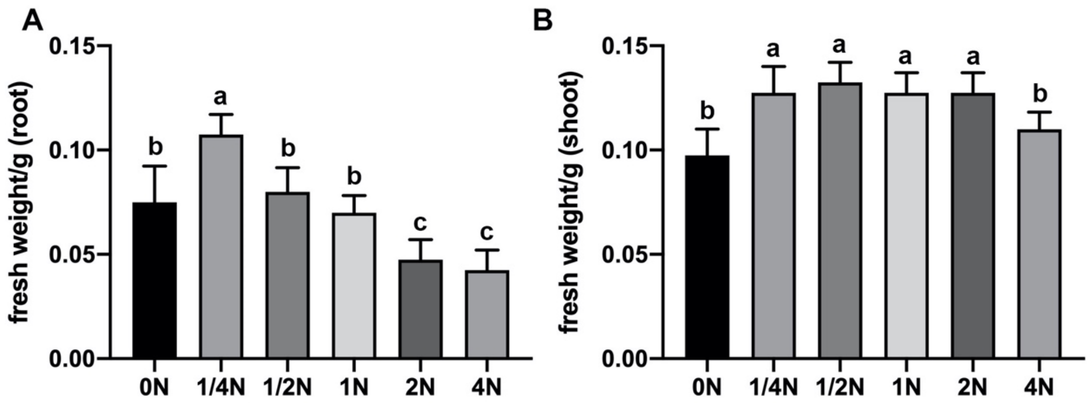

Supplementary Figure S1. Fresh weight (g) of the LY9348 in the roots (**A**) and in the shoots (**B**) grown hydroponically for 14 days at six N concentrations. Bars represented the standard error (SE; n=3). Different letters indicate significant differences (P < 0.05, one-way ANOVA, Tukey's HSD test).

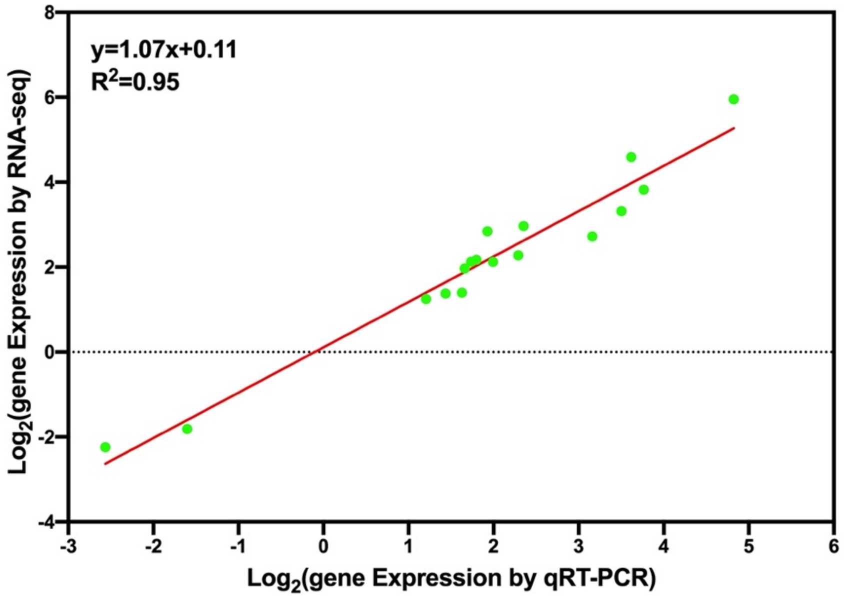

Supplementary Figure S2. Correlation analysis between qRT-PCR gene expression level and corresponding RNA-Seq data. **: p<0.01.

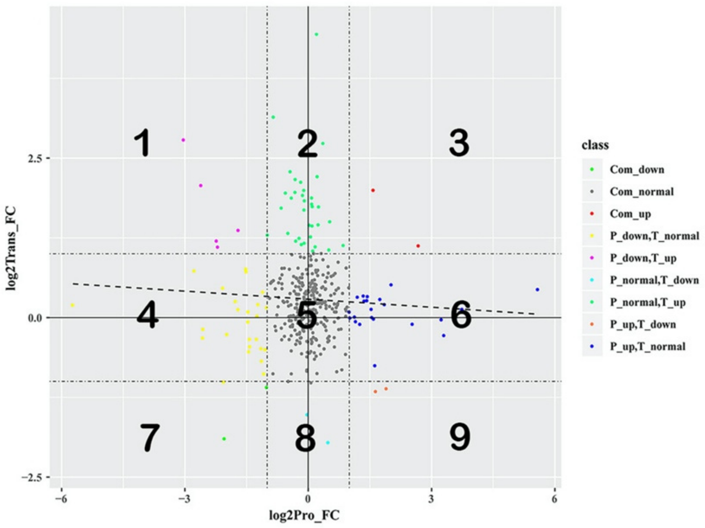

Supplementary Figure S3. Correlation diagram of expression ratio between transcriptome and proteome. The ordinate represented $\log_2$FC value of DEGs, and the abscissa represented $\log_2$FC value of DAPs

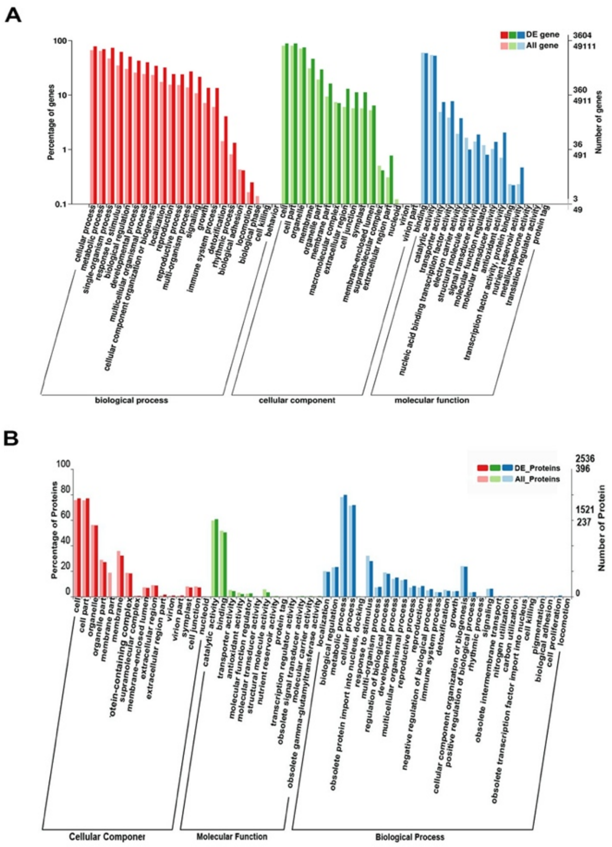

Supplementary Figure S4. Comparative analysis of GO annotation between DEGs and DAPs. (**A**) Functional categorization of DEGs between 1/4N and 1N; (**B**) Functional categorization of the DAPs between 1/4N and 1N. GO: Gene Ontology. DEGs: different expression genes; DAPs: different abundance proteins.

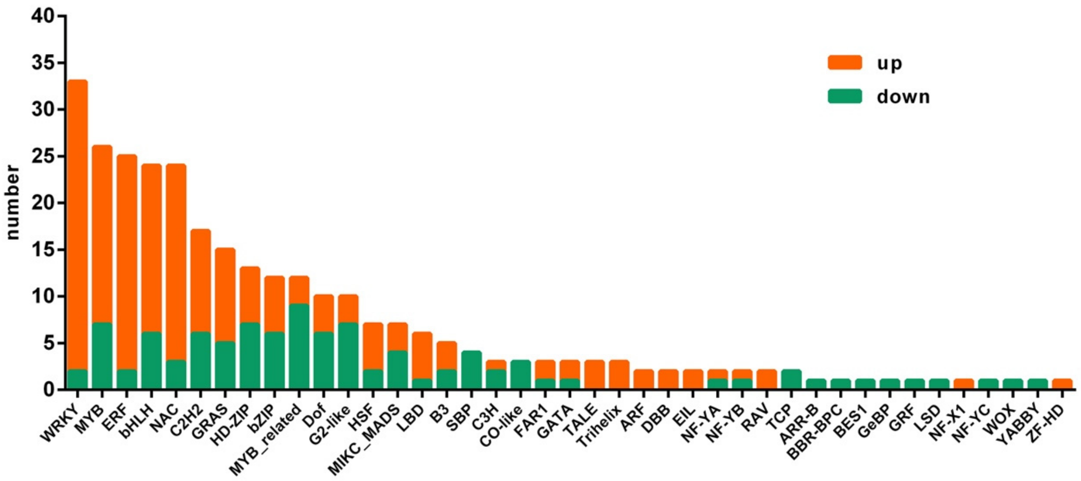

Supplementary Figure S5. The transcription factors (TFs) classification of DEGs analyzed through PlantTFDB website.

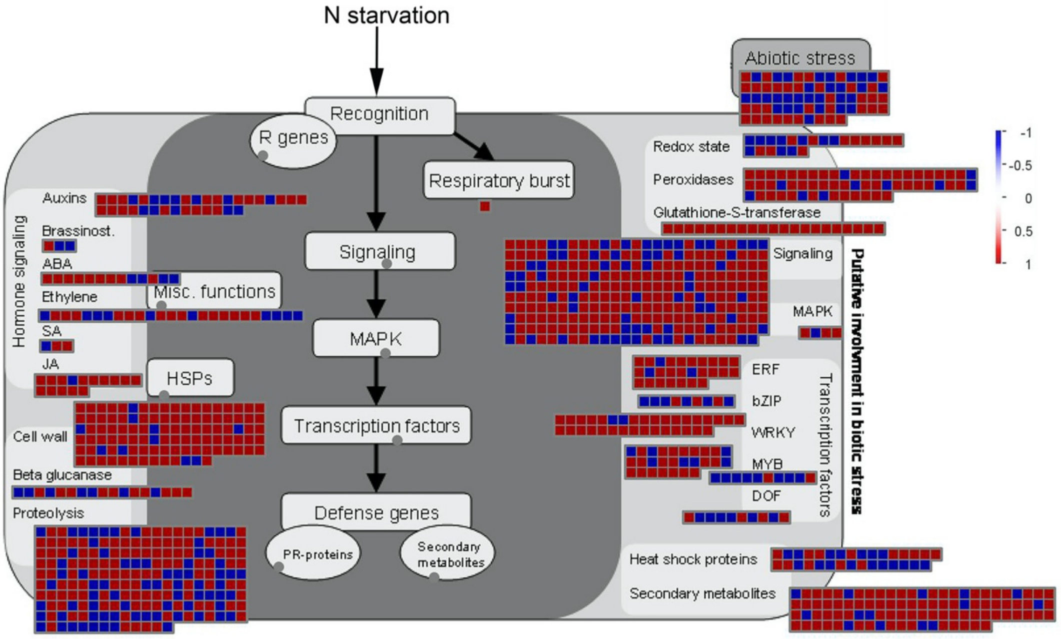

Supplementary Figure S6. The model of the regulation mechanism of rice roots in response to N starvation involves numerous pathways by MapMan software. Small squares represented DEGs. Red/blue indicated that gene expression level was induced/repressed compared with the control (1 N).
