# Supplementary material for: Integrative Transcriptomic and Proteomic Analysis Reveals an Alternative Molecular Network of Glutamine Synthetase 2 Corresponding to Nitrogen Deficiency in Rice (Oryza sativa L.)
Source: Int J Mol Sci. 2021 Jul 18;22(14):7674. doi: 10.3390/ijms22147674 (PMC8304609; doi:10.3390/ijms22147674)
Supplement: Supplementary file 1 [file ijms-22-07674-s001.zip › ijms-1238115-supplementary/Supplementary files/Supplementary table 11.pdf]

Supplementary Table S11. Primers of mRNAs used in this study.

Primer ID	Forward primers (5'to3')	Reverse primer (5'to3')
OsNRT2.1	CTTCACGTCGTCGAGGTAC	CACTCGGAGCCGTAGTAGTG
OsNRT2.2	CATCGCCGATACTTCTAC	ATCCAAATGTTCCAGAGGCG
OsNRT2.3a	CGCTGCTGCCGCTCATCCG	CCGTGCCCATGGCCAGAC
OsNRT2.4	CTCACCTTCGGCATCGTTCCCTT	CGTGTACCTGGACCCGCTGAAGA
OsNAR2.1	CTCGTTGGTGCTCGTCTTGCTCG	GCACAGCTTCACCTTCACGCTCTTG
OsAMT1.3	TACGACTACGACTTCTTCCTCTTCC	GGTGAGGAAGGCGGAGTAGATGAGA
OsAMT3.3	ACCAGACCACTCCGTCATCG	CGCCGAGTTGATAGCCCAC
OsAMT1.2	GCTTCGACTACAGCTTCTTCCTCTT	GGTGAGGAAGGCGGAGTAGATGAGG
OsNR2	GCTGGTTCAGGGTGAAGACGAAG	TTGAGGAACGGCGTGGACGTGCTGC
carbonic anhydrase	GGATGGGTTCGCCAAGTTCA	GCTTGATCTTGCAGTAAGCTGGGAC
OsNPF6.1	TACAGGAAGCGTCACATCAAGCACA	GCCATGGGTTATTGATTTCTTCTTG
OsNPF4.1	CTCTTCGCCGTCACCTTCTCCA	GGTAGAAGCCGAAGGAGTAGGAGCAGT
OsNADH-GOGAT2	TCACGAAGGACGCAGGTT	CCAAGCCCGAGTTGTCTG
OsGS1; 1	CACGAGACCGCCGACATCAA	ACGATGTAAGGGTCCATGTTGGACG
OsGS1; 2	AGACCGCCGACATCAACACCT	CTGTCCTCGAAGTACCCCTTGCCCT
OsGS1; 3	ATTGCTGGCGTCGTCGTGT	TGTTGATGTCGGCGGTCTCG
OsAS1	ATGTGCGGGATCCTGGCCGTG	GCCGGTGCTGAACGTGTGCTTG
