# Supplementary material for: Integrative Transcriptomic and Proteomic Analysis Reveals an Alternative Molecular Network of Glutamine Synthetase 2 Corresponding to Nitrogen Deficiency in Rice (Oryza sativa L.)
Source: Int J Mol Sci. 2021 Jul 18;22(14):7674. doi: 10.3390/ijms22147674 (PMC8304609; doi:10.3390/ijms22147674)
Supplement: Supplementary file 1 [file ijms-22-07674-s001.zip › ijms-1238115-supplementary/Supplementary files/Supplementary Table 3.pdf]

Supplementary Table S3. KEGG pathways associated with the number of up-regulated and down-regulated DAPs.

Pathways	P value	Pathway ID	Up regulated	Down regulated
Phenylpropanoid Biosynthesis	0.2502138	ko00940	6	11
Phenylalanine Metabolism	0.1485072	ko00360	6	10
Oxidative phosphorylation	0.8849044	ko00190	6	8
Glycolysis/Gluconeogenesis	0.6223506	ko00010	7	6
Starch and Sucrose Metabolism	0.3519892	ko00500	3	10
Glutathione Metabolism	0.1692994	ko00480	7	5
Alanine, Aspartate and Glutamate Metabolism	0.0839540	ko00250	5	5
Arginine and Proline Metabolism	0.1501081	ko00330	5	4
Ubiquinone and Other terpenoid-quinone biosynthesis	0.0013467	ko00130	3	5
Fructose and Mannose metabolism	0.3569871	ko00051	2	5
Pyruvate Metabolism	0.8653597	ko00620	5	2
Carbon fixation in Photosynthetic organisms	0.8342573	ko00710	2	4
Galactose Metabolism	0.2388235	ko00052	1	5
Glycine, Serine and Threonine metabolism	0.5047794	ko00260	2	4
Purine Metabolism	0.7693221	ko00230	3	3
Ubiquitin Mediated Proteolysis	0.3256393	ko04120	2	4
Pentose phosphate pathway	0.8224658	ko00030	0	5
Porphyrin and Chlorophyll Metabolism	0.0116692	ko00860	3	2
Pyrimidine Metabolism	0.5171418	ko00240	2	3
Tropane, Piperidine and Pyridine Alkaloid Biosynthesis	0.0398541	ko00960	4	1
Tyrosine Metabolism	0.4194729	ko00350	3	2
Amino sugar and nucleotide sugar Metabolism	0.8549613	ko00520	2	2
Ascorbate and Aldarate Metabolism	0.7233889	ko00053	2	2
Flavonoid Biosynthesis	0.7842114	ko00941	2	2
Glyoxylate and Dicarboxylate Metabolism	0.4602095	ko00630	1	3
Lysine Biosynthesis	0.1040697	ko00300	2	2
N-Glycan Biosynthesis	0.0787221	ko00510	1	3
Nitrogen Metabolism: Reduction and Fixation	0.3190856	ko00910	2	2
Propanoate Metabolism	0.5666894	ko00640	4	0
Butanoate Metabolism	0.6619827	ko00650	2	1
Fatty Acid Metabolism	0.8388311	ko00071	2	1
Selenoamino acid Metabolism	0.5913148	ko00450	0	3
Carotenoid Biosynthesis	0.4691522	ko00906	1	1
Indole Alkaloid Biosynthesis	0.1180905	ko00901	1	1
Linoleic Acid Metabolism	0.7849148	ko00591	1	1
Sulfur Metabolism: Reduction and Fixation	0.5333963	ko00920	1	1
Folate Biosynthesis	0.8713562	ko00790	0	1
Nicotinate and Nicotinamide Metabolism	0.6407041	ko00760	1	0
Sulfur Relay System	0.4003264	ko04122	1	0

DAPs: differential abundance proteins.
