# Supplementary material for: Integrative Transcriptomic and Proteomic Analysis Reveals an Alternative Molecular Network of Glutamine Synthetase 2 Corresponding to Nitrogen Deficiency in Rice (Oryza sativa L.)
Source: Int J Mol Sci. 2021 Jul 18;22(14):7674. doi: 10.3390/ijms22147674 (PMC8304609; doi:10.3390/ijms22147674)
Supplement: Supplementary file 1 [file ijms-22-07674-s001.zip › ijms-1238115-supplementary/Supplementary files/Supplementary Table 6.pdf]

Supplementary Table S6. A list of the selected DEGs for qRT-PCR validation.

#ID	1N_FPKM	1/4N_FPKM	log_2FC	regulated	NR_annotation
LOC_Os02g02170	22.055874	197.19722	3.31747201	up	*OsNRT2.1*
LOC_Os02g02190	3.236936	181.750443	5.95118443	up	*OsNRT2.2*
LOC_Os01g50820	16.658255	361.171936	4.59409704	up	*OsNRT2.3a*
LOC_Os01g36720	0.283683	2.289304	2.96842927	up	*OsNRT2.4*
LOC_Os02g38230	52.952381	671.419495	3.82136082	up	*OsNAR2.1*
LOC_Os02g40710	2.699628	17.60108	2.84038042	up	*OsAMT1.3*
LOC_Os02g40730	21.85079	88.213524	2.1700885	up	*OsAMT1.2*
LOC_Os02g34580	0.222772	1.654249	2.72499263	up	*OsAMT3.3*
LOC_Os02g53130	11.642644	27.493874	1.3973264	up	*OsNR2*
LOC_Os01g45274	74.204513	172.73428	1.37811461	up	carbonic anhydrase
LOC_Os01g01360	4.784554	1.196574	-1.8131084	down	*OsNPF6.1*
LOC_Os11g12740	23.039596	4.369908	-2.2402866	down	*OsNPF4.1*
LOC_Os05g48200	0.061253	0.317122	2.27828493	up	*OsNADH-GOGAT2*
LOC_Os02g50240	53.83755	210.194171	2.12672899	up	*OsGS1; 1*
LOC_Os03g12290	121.597572	426.037476	1.96788266	up	*OsGS1; 2*
LOC_Os03g50490	1.723211	7.012081	2.12211271	up	*OsGS1; 3*
LOC_Os03g18130	66.694927	140.419938	1.24775866	up	*OsAS1*
